# Fertile fathoms: Deep reproductive refugia for threatened shallow corals

**DOI:** 10.1038/srep12407

**Published:** 2015-07-21

**Authors:** Daniel M. Holstein, Tyler B. Smith, Joanna Gyory, Claire B. Paris

**Affiliations:** 1 Marine Biology and Fisheries, Rosenstiel School of Marine and Atmospheric Science, University of Miami, Miami, FL 33149, USA; 2 Center for Marine and Environmental Studies, University of the Virgin Islands, St. Thomas, VI 00802-9990, USA; 3 Department of Ecology and Evolutionary Biology, Tulane University, New Orleans, LA 70118, USA; 4 Department of Ocean Sciences, Rosenstiel School of Marine and Atmospheric Science, University of Miami, Miami, FL 33149, USA

## Abstract

The persistence of natural metapopulations may depend on subpopulations that exist at the edges of species ranges, removed from anthropogenic stress. Mesophotic coral ecosystems (30–150 m) are buffered from disturbance by depth and distance, and are potentially massive reservoirs of coral diversity and fecundity; yet we know little about the reproductive capabilities of their constituent species and the potential for these marginal environments to influence patterns of coral reef persistence. We investigated the reproductive performance of the threatened depth-generalist coral *Orbicella faveolata* over the extent of its vertical range to assess mesophotic contributions to regional larval pools. Over equal habitat area, mesophotic coral populations were found to produce over an order of magnitude more eggs than nearby shallow populations. Positive changes with depth in both population abundance and polyp fecundity contributed to this discrepancy. Relative larval pool contributions of deeper living corals will likely increase as shallow habitats further degrade due to climate change and local habitat degradation. This is a compelling example of the potential for marginal habitat to be critical to metapopulation persistence as reproductive refugia.

Reproductive performance can influence patterns of population persistence across a species’ range, and thus, understanding how reproductive performance changes across environmental gradients is central to the study of evolutionary ecology and to the successful management of natural populations and threatened species^1^. Species range is often used as a biogeographic term, describing the large scale limits of a species distribution over the two-dimensional surface of the earth, and often in reference to latitudinal clines^2^,^3^. The conventional understanding, termed Brown’s Principle, suggests that favorable environmental conditions near the center of a species’ biogeographic range results in higher individual reproductive success and, subsequently, higher population abundance than towards the edges of a biogeographic range^4^. However, not all species distributions follow this “abundant center” pattern, with many showing higher densities towards a range margin, or heterogeneous densities throughout the range^5^,^6^,^7^. Additionally, high adult abundances may not always be coupled with high individual performance and reproductive capacity^8^. This may be particularly true in marine species that possess a pelagic larval phase capable of dispersing larvae over large distances, and for which population densities may be influenced more by oceanographic conditions affecting connectivity and recruitment than by local environmental conditions and adult reproductive performance^7^,^9^.

For many species, biophysical characteristics that influence fitness and distribution can vary more dramatically over short vertical distances, such as elevation or depth, than they do over large horizontal distances. Again, there may be a supposition that individual performance should be maximum near the centers of vertical species ranges^4^,^10^. However, Brown’s Principle is not always immediately intuitive over vertical habitat ranges, particularly in the ocean where species distributions may be bounded by the sea surface, and resources such as light are maximum at this boundary. In light dependent corals it could be expected that individual success wanes at the deeper ends of species vertical ranges where critical resources, such as light, are more limiting. Yet we find that population abundance may be greatest at the deepest margins of the range in some light dependent coral species, such as *Orbicella* spp.^11^,^12^,^13^,^14^, and bimodal depth-related growth maxima have been observed in other depth-generalist species^15^.

Exceptions to Brown’s Principle may be of particular importance to coral reef persistence, as individuals at the edge of a species’ vertical range may become proportionately more important in declining populations. Shallow coral reefs are at escalating risk of habitat degradation^16^,^17^,^18^, yet mesophotic coral ecosystems (MCEs) between 30 and 150 meters depth are buffered from many stressors, particularly coral thermal bleaching^19^,^20^,^21^. This observation, and the considerable overlap of scleractinian coral species between MCEs (30–50 m) and shallow reefs (<30 m) in the Caribbean (~70%;^14^), has given rise to the Deep Reef Refugia Hypothesis (DRRH)^22^,^23^, that posits a depth refugia for shallow water coral species and a stable pool of reproductively capable corals that could assist in shallow water recovery, thus increasing resilience after disturbance.

We know little about coral reproduction at MCE depths, including whether MCE corals are reproductively competent, how their reproductive effort changes with depth, and whether that reproductive effort results in successful recruitment or larval exchange^22^. The little available information largely suggests reduced fecundity with increasing depth. For example, in some brooding corals reproductive output decreases with depth, and this is likely due to phototrophic energy limitations^24^,^25^; however, this relationship may not be consistent for all brooding species in all locations^26^. Information regarding depth-fecundity relationships in broadcasting species is extremely rare and has not been accomplished in mesophotic depths, though no change in polyp-specific gamete production in the broadcasting species *O. faveolata* was found over the first half of its depth range (3–18 m)^27^. A decline in reproductive output with depth could suggest minimal MCE contributions to regional larval pools and diminish the validity of the DRRH. However, the high abundance of depth generalist coral species in MCE reefs combined with the observation that the extent of known MCEs is large^14^ and in some areas may surpass the total area of shallow water reefs (authors, unpub. data), could suggest high reproductive output despite potential light limitations. This may be especially true if energetic constraints are relaxed by increasing heterotrophy^15^,^28^. Indeed, there is potential that MCEs produce more coral gametes than shallow reefs depending on how abundance and reproductive capacity vary with depth.

Our study resolves the reproductive uncertainty over depth for the important—and recently listed as threatened^29^—Caribbean broadcast spawning coral *Orbicella* spp. in the US Virgin Islands (USVI) (Fig. 1) and makes estimates of depth-specific contributions to regional reproductive output. Unlike previous studies that have examined coral fecundity and depth at the scale of individual polyps or colonies, the current study expanded these parameters to make realistic estimates of the relative egg production of *O. faveolata* across its depth range by scaling ova production by population estimates. Our findings suggest that complex interacting environmental gradients and disturbance regimes complicate the application of Brown’s Principle in vertical marine environments, and challenge the assertion that coral growth optima and light saturation correlate to fecundity optima. Here we reaffirm the potential importance of mesophotic coral reefs as larval resources in regional metapopulations.

## Results

### Gametogenesis and fecundity

*O. faveolata* colonies sampled from three depths—shallow (5–10 m), mid-depth (15–22 m) and mesophotic (35–40 m) (n ≥ 25 over five weeks)—were found to contain both male and female gametes, which were visually indistinguishable by depth (Fig. 2). Spermaries of stages II-V and oocytes/ova of stages II–IV^30^ were identified in corals from all sites (Fig. 2 and Supplementary Fig. S2). Stage I spermaries and oocytes were also observed at all sites; however, they are not included in this analysis due to difficulty in identification of these early stage gametes. Analysis of spermatogenesis suggested development from earlier stage spermaries in late July and early August to late stage mature spermaries prior to spawning at all sites (Fig. 3). Late stage spermaries were absent (lost) from coral tissues at all sites one week after full moon, which indicated these populations had spawned. In some cases, remnant stage V spermaries were observed with free spermatozoa in the mesentery, which is evidence of spawning behavior, and suggests these spermaries were residual after spawning occurred (the presence of remnant sperm is thus not represented in Fig. 3, also see Supplementary Fig. S2). Specifically, at the mesophotic site 60% of colonies contained stage V spermaries on the 19^th^ of August, and by the 25^th^ of August no colonies contained intact stage V spermaries. Both shallow and mid-depth sites experienced similar losses of stage V spermaries from 40% of colonies. Additional evidence of spawning was present in tissues from all sites in the last week of sampling, including wasted mesenteries. The presence of early stage spermaries increased in colonies at all sites late in August, suggesting a second spawn in September (Fig. 3).

By the first week of sampling in 2011 (just before new moon) over 75% of colonies from each site contained stage II oocytes (Fig. 3). Colonies rapidly lost stage II oocytes as they developed into stage III and IV oocytes/ova throughout the sampling period. This development was delayed but more rapid in mesophotic colonies (Figs 3 and 4). Histological evidence of spawning characterized by the loss of stage IV ova from coral tissue was seen in mesophotic colonies, of which 100% contained stage IV ova just prior to the time of expected spawning, and 60% contained stage IV ova one week later. A loss of stage IV ova was also observed in shallow colonies during this time. There was evidence of ova retention and development beyond the date of August 2011 spawning at all sites, again suggesting an additional September spawn.

Oocyte/ovum diameters were measured from ~3 random oocytes/ova in each of ~3 gravid polyps per colony. Oocyte diameters increased during the first three weeks of sampling at all sites. During weeks two and three, oocytes measured from mesophotic corals were significantly smaller than those from shallow corals, and significantly smaller than those from mid-depth corals in week two (multiple comparisons from linear mixed effect models within weeks, step-wise adjusted; see Table 1 for more information and exact adjusted p-values) (Fig. 4). However, by week four there were no significant differences in oocyte/ovum diameter between sites, and the same was true for week five, post August spawning. At week four, mean oocyte/ova diameters (±SD) for shallow, mid-depth and mesophotic corals were 283.36 μm (61.93), 265.77 μm (59.22), and 275.01 μm (45.60), respectively.

Fecundity per polyp was estimated as the product of the average number of gonads per polyp in cross-section and the average number of oocytes or ova per gonad in longitudinal section for 2–3 polyps per orientation per sample. The number of gonads per polyp, the number of oocytes/ova per gonad and the product of these two estimates were pooled between weeks 1 through 4 (pre-spawning samples). The pooled results were non-normal after log-transformation (Levene Tests, p > 0.5). The number of gonads per polyp is generally 12 (one gonad per septa), however mesophotic colonies had significantly more gonads per polyp than either shallow or mid-depth corals (Kruskal-Wallis ANOVA/Bonferroni method, p = 0.0028; post-hoc comparison, mesophotic > mid-depth and mesophotic > shallow, p = 0.001 and 0.033, respectively) (Table 2, Fig. 5). Polyps from all sites were sometimes found to have as many as 24 gonads (two gonads per septa). The number of oocytes/ova per gonad generally ranged from 8–12 (maximum recorded was 19), however polyps in mesophotic corals had significantly more oocytes/ova per gonad than shallow corals (Kruskal-Wallis ANOVA/Bonferroni method, p = 0.046; post-hoc comparison, p = 0.028) (Table 2, Fig. 5 and Supplementary Fig. S3). There was no difference found in oocytes/ova per gonad between mesophotic and mid-depth colonies, or between mid-depth and shallow colonies.

The product of gonads per polyp and ova per gonad provided an estimate of polyp fecundity, which was found to be heterogeneous across sites, with mesophotic corals having significantly higher oocyte/ovum production per polyp than shallow corals (Kruskal-Wallis ANOVA/Bonferroni method, p = 0.021; post-hoc comparison, p = 0.009) (Table 2, Fig. 5). Again, no difference was found between mesophotic and mid-depth colonies, or mid-depth and shallow colonies; however, there was a significant increasing linear relationship between fecundity and depth (p < 0.001, R^2^ = 0.18, Fig. 6a). No significant relationship was found between oocyte/ovum production per polyp and the surface area of sampled colonies.

### Reproductive capability

The third order polynomial model fitted to *Orbicella* spp. coral cover versus depth (p < 0.0001, R^2^ = 0.51, Fig. 6c) indicates that coral cover increases with depth nonlinearly until ~30 m where it stabilizes at ~28% until a depth of ~40 m where coral cover begins to decline dramatically. By 50 m depth *Orbicella* spp. coral cover drops to zero. Shallow corals had significantly higher numbers of polyps per cm^2^ (on average, nearly twice) compared to both mid-depth and mesophotic corals, which were not significantly different from each other (one-way ANOVA, p = 0.012). The same data was log-transformed and a linear regression with depth was performed (p = 0.014, R^2^ = 0.381, Fig. 6b).

The resulting regression models from the previous three analyses (oocyte/ovum production per polyp, coral cover, and the number of polyps per cm^2^) were used to estimate the number of ova produced within a hypothetical 1 km^2^ reef area with increasing depth from 10–50 m (equation 2, Fig. 6). For comparison, an additional scenario assuming equal ova production at all depths was also estimated (Fig. 6d).

Ova production is estimated to be an order of magnitude greater at 35 m than at 10 m (1.146*10^12^, or over 300% more ova per km^2^). Higher fecundity in mesophotic corals resulted in 41% (443 billion) more ova produced per km^2^ at 35 m than a scenario assuming equal fecundity. Thus, higher mesophotic coral cover accounts for 163.5% (667 billion) more ova produced per km^2^ at 35 m compared to at 10 m.

### Spawning observations

Video observations in the field in August and September 2012 showed mesophotic spawning in *Orbicella* spp. On August 11^th^, 9 nights after full moon, spawning was observed in an *O. faveolata* colony at 38 m at ~21:00. In addition, gamete bundles were observed in the water column. On the evening of September 7^th^ (7 nights after full moon), a full-colony spawn of *O. franksi* was captured on video at ~38 m at 20:49 (Supplementary Fig. S1).

Spawning behavior was also observed in *O. faveolata* partial colonies kept in the laboratory. On the evening of August 10^th^ (8 nights after full moon) one mesophotic colony of ten released gamete bundles at ~21:00, and one shallow colony of ten released gamete bundles about 2 hours later at ~23:00. These were not whole-fragment spawns. On September 7th (7 nights after full moon) four of ten mesophotic colonies and three of ten shallow colonies released gamete bundles in whole-fragment spawns. Mesophotic colonies released gametes between 20:00 and 20:45, whereas shallow colonies released gametes between 21:45 and 22:30, with “dribbles” occurring as late as 23:20.

## Discussion

Depth provides a reproductive refuge for a threatened depth-generalist coral species in the Caribbean. Our study suggests that MCEs in the USVI have far greater orbicellid gamete output per area than nearby shallow water reefs, despite reduced solar radiation and contrary to established relationships for some brooding corals. USVI orbicellid abundance and reproductive capacity throughout its vertical range appears to be an exception to Brown’s Principle, and USVI MCEs may be a compelling example of poorly understood marginal habitat that is crucial to population persistence. By resolving the depth-fecundity relationship for *O. faveolata*, we have addressed an information gap in evaluating the potential for MCEs to serve as refugia for this species^22^,^23^,^31^.

A novel result of this study was that mesophotic *O. faveolata* coral polyps were in fact more fecund than shallower corals, and that fecundity increased linearly with depth. No differences in the number of gonads per polyp or the number of oocytes per gonad were found between shallow (5–10 m) and mid-depth (15–22 m) sites, corroborating previous findings^27^, and in these depths *O. faveolata* fecundity estimates fell within the ranges recorded by previous studies^27^,^32^,^33^,^34^. However, the highest estimated fecundities in this study—all from MCE corals—nearly doubled previously recorded maximum values, and fecundity estimates were significantly greater in MCE corals than in shallow corals.

There are three potential explanations for the less than intuitive depth-fecundity relationship found in this study that appears to violate Brown’s Principle. First, it is possible that environmental conditions conducive to reproductive energy allocation in this species are decoupled from light saturation. Energy may not be limiting at depth, as assumed based on the sharply attenuated light fields in mesophotic environments. The Caribbean coral *Madracis mirabilis* showed bimodal growth in Jamaica, with peaks in shallow water (10 m) and at mesophotic depths (30 m)^15^. The latter peak in growth corresponded with the summer chlorophyll maximum layer depth at the first thermocline, which may have offered a consistent source of heterotrophically derived energy. Although heterotrophy has been shown to be insufficient to meet total metabolic needs in shallow *O. faveolata*^35^, mesophotic *Orbicella* spp. in the USVI are associated with the chlorophyll maximum layer before and during reproductive periods and bottom currents can be stronger than in shallow water habitats, potentially increasing advective food supply^14^. In addition, temperatures are lower in mesophotic environments by 1 °C or more during reproductive periods^14^, which could lower metabolic demands and allow more investment in reproduction. Thus, *Orbicella* spp. in mesophotic environments of the Caribbean may be less energetically limited than shallow water corals, which could enable them to allocate relatively more energy into reproduction. The same may apply to other coral species in mesophotic environments associated with the first thermocline.

A second non-exclusive hypothesis is that the depth-fecundity relationships seen in *O. faveolata* may have to do with differential disturbance regimes across depths in the USVI. The relatively low disturbance and stress experienced by mesophotic coral reefs^12^,^13^,^23^,^31^,^36^ may allow corals entering reproductive phases to divert energy otherwise allocated for growth and colony maintenance. As a terrestrial corollary, some plants are capable of separating periods of growth from periods of fruiting when in stable, low variability environments in order to maximize reproductive potential, with ‘seed years’ in trees being a particularly good example^37^. It is possible that low disturbance and stress in USVI mesophotic environments—due to relatively consistent solar irradiation and temperature, low thermal bleaching incidence and reduced storm damage—results in the temporary cessation of growth and tissue maintenance and near-maximum gamete production during *O. faveolata* reproductive periods. Indeed, in 2010 shallow water *Orbicella* spp. in the USVI experienced a mean of 4.1 (±0.7 SEM, n = 8 sites) degree heating weeks and an elevated prevalence of stark white bleaching of 48.2% (±6.8, n = 10), whereas mesophotic corals experienced 0.1 (±0.1, n = 4) degree heating weeks and a non-elevated bleaching prevalence of 16.0% (±3.2, n = 4) (authors, unpub. data) which potentially contributed to the discrepancies observed in shallow and deep coral reproductive capacity in the following year. Nonetheless, measured fecundities close to published literature values in shallow colonies suggest that shallow water stress in 2010 could not explain all differences observed between depths.

A third explanation for higher fecundity in MCEs, which opposes the first two hypotheses, is that variation in habitat quality in mesophotic ecosystems increases adaptive pressure on reproductive synchrony, reproductive energy allocation and dispersal^38^. Contrary to the conventional understanding that physiological stress reduces reproductive output^39^, physiological stress encountered at the limits of a species’ range may in some instances induce increases in reproductive output^7^,^10^, suggesting the potential for adjustments in phenotypes. While many types of physiological stress and disturbance may be reduced in MCEs, the fact that the MCE orbicellids are at the maximum depth of their vertical range suggests a hard physiological barrier to survival, such as photosynthetic energy contribution at reduced light. Additionally, the delayed but rapid development of oocytes observed in mesophotic *O. faveolata* histological sections may imply that mesophotic colonies that have entered a reproductive phase divert a greater proportion of metabolic energy to reproduction than do shallow colonies. If this is the case, mesophotic *O. faveolata* may be particularly vulnerable to mortality from disease or physical damage during or directly after reproductive cycles due to energetic limitations akin to those experienced by corals after bleaching stress^40^,^41^.

Video collected in the field and laboratory in 2012 suggests synchronous spawning on the order of hours and days of *O. faveolata* at sites separated by ~11 km horizontally and ~30 m vertically. Mesophotic *O. faveolata* colonies spawned earlier (~1 hr) than shallow colonies when observed in the laboratory. Although laboratory conditions may skew spawning synchrony due to altered light cycles and separation from conspecifics, earlier spawning in deeper-living corals may also be due to truncated daylight hours at depth and an earlier sunset cue.

Histological analysis of gametogenesis and direct observations of *O. faveolata* spawning behavior support the assertion that spawning at shallow, mid-depth and mesophotic sites is synchronous within days, if not hours. It is unlikely that direct hybridization occurs between mesophotic and shallow *O. faveolata* colonies in the Northern USVI, despite the potential for synchronous gametogenesis and spawning behavior. Considerable horizontal distances separate the habitats in most cases and it is not likely that unfertilized gametes would meet. However, hybridization could be likely on wall or seamount habitats^42^, where buoyant or swimming gametes may have a greater potential of fertilization with conspecifics from a wide range of depths. The potential for deep and shallow hybridization in different habitat types warrants future investigation, and has implications for population connectivity and rates of coral adaptation, speciation and evolution.

Assuming equal habitat area, it appears that over 85% of *Orbicella* spp. (and *O. faveolata* specifically) ova in the USVI are produced below 20 m, and over 50% are produced between 30 m and 50 m, in MCEs. However, this likely underestimates mesophotic contributions in the USVI. A large MCE south of St. Thomas has been characterized^13^,^14^, where coral habitat extent is nearly half the total shallow coral reef extent in St. Thomas and St. John combined (mapped by the National Oceanic and Atmospheric Administration’s Center of Coastal Monitoring and Assessment). The true extent of MCEs in the USVI, and indeed in the Caribbean basin, is unknown, but there is potential that MCEs rival or surpass shallow reefs in habitat area^43^, which would imply even greater larval contributions from MCEs than suggested here.

In addition, the relative contribution of mesophotic larvae to regional larval pools could be increasing with shallow water reef degradation. Shallow reefs in the USVI and Puerto Rico experienced a nearly 10% absolute loss of coral cover between 1975 and 2000^44^, and an additional relative 48%–61.8% loss in coral cover in shallow reefs after the 2005 bleaching and disease event^40^,^45^. The impacts on shallow water coral cover were greatest in *Orbicella* spp.^45^. USVI MCEs and mesophotic orbicellids appear to have been spared this fate^14^, and growing disparity in coral cover between shallow and mesophotic coral reefs suggests that the proportion of all coral reef larvae produced in MCEs will continue to grow.

Fertilization—a crucial step in the reproductive cycle—was not addressed in this study. The physical conditions of deep and shallow environments may create disparate fertilization conditions for corals in a number of ways, the most obvious being for broadcast spawning species such as *O. faveolata*. Traditionally it is understood that *O. faveolata* gamete bundles rise to the air-sea boundary where fertilization occurs after they are concentrated and break apart. Depth, therefore, may limit fertilization success in this species, as gamete bundles may break apart, disperse or be preyed upon before they reach the sea surface. Understanding the effects of depth on fertilization rates of corals is important for accurately estimating larval load, and will be the focus of future research.

Similarly, larval survivorship and post-settlement mortality have implications for the refugia potential of MCEs. For example, *Agaricia agaricites* larvae taken from different depths show differential survivorship when exposed to UVB radiation^46^. Larvae of mesophotic origin may have different rates of pelagic and post-settlement mortality as compared to larvae from shallower habitats, which must be quantified if we hope to understand how MCEs contribute to coral reef resilience through larval exchange. Studies suggest that both coral and endosymbiont genetic connectivity vary by location and by species, and in some species speciation may be occurring across depth^47^,^48^,^49^,^50^; thus, refugia habitat will likely require a suite of overlapping protective characteristics as well as larval exchange with adjacent habitats.

The dispersal of orbicellid larvae between MCEs and shallower habitats was not addressed in this study, however study of *Montastraea cavernosa*—another broadcasting species—in the USVI has suggested that genetic mixing across depth may occur^50^, and biophysical modeling suggests that vertical migration of orbicellid larvae between coral reefs in the USVI is possible^51^. In order for MCEs to behave as functional refugia and contribute to coral reef resilience, they must contribute to the demography of coral reefs in general, and more study of how these systems interact and exchange larvae with adjacent habitats is necessary. As shallow coral habitats continue to be degraded and fragmented, MCEs may prove to be more and more important as potential sources of coral reef larvae, and it is crucial that these habitats be given adequate conservation attention despite being located at the limits of many species ranges. Continued evaluation of the potential for mesophotic reproductive refugia to provide coral reef larvae to both shallow and mesophotic environments will aid in discerning the complex roles these marginal—but not negligible—coral reefs have played, do play and will play in Caribbean coral reef metapopulations.

## Methods

**Figure 1:**
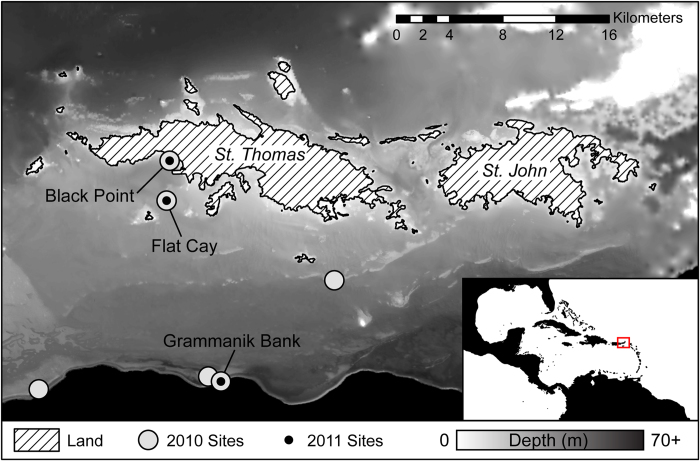
The northern US Virgin Islands of St. Thomas and St. John. Considerable mesophotic habitat (30–150 m) exists on the broad insular platform, and well-mapped linear coral habitat exists on submerged banks near the shelf edge south of St. Thomas^11^,^13^,^14^. Sample sites from 2010 (gray) ranged in depth from 6–43 m. In 2011 a subset of 2010 sites were visited weekly for five weeks bracketing spawning in August: Black Point (5–10 m), Flat Cay (15–22 m), and Grammanik Bank (35–40 m). Map created using ArcGIS 10.

**Figure 2:**
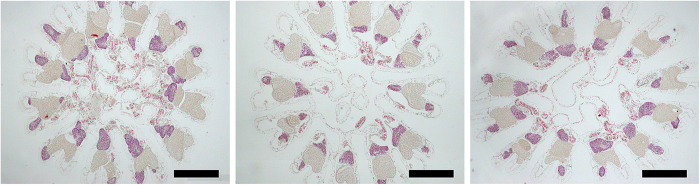
Histological cross-sections of fully fecund *O. faveolata* polyps just prior to spawning (week 4) from each site. From left to right, in order of descending depth, Black Point (8 m), Flat Cay (19 m) and Grammanik Bank (39 m). In each example the polyp has at least 12 ripe gonads, all ova are stage IV (stained gold), and spermaries are stage V (stained red). Bar = 500 μm. See Supplementary Fig. S2 and Supplementary Fig. S3 for further reproductive structure identification.

**Figure 3:**
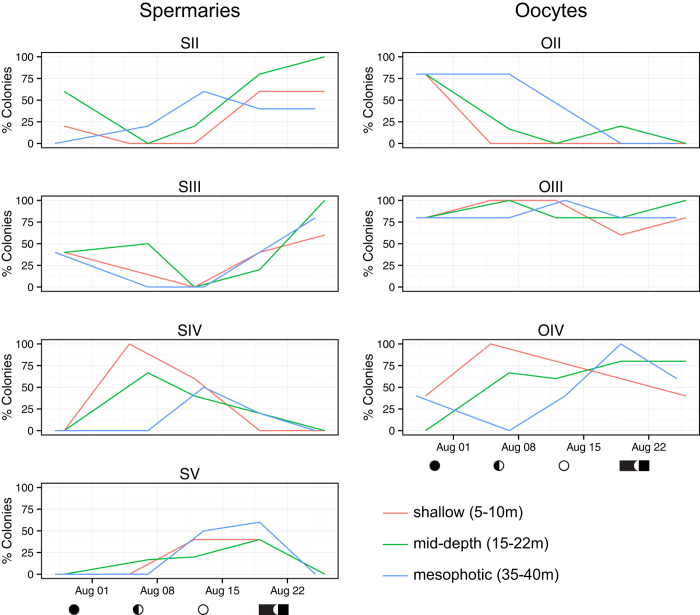
*O. faveolata* gametogenic stages of spermaries (left column) and oocytes (right column) observed in histological sections collected weekly (July 28^th^ to August 26^th^ 2011, 5 sampling times) from three sites: a shallow near-shore site (red); an offshore island mid-depth site (green); and a mesophotic submerged bank site (blue). Gametocytes were staged as I–V for spermaries and I–IV for oocytes^33^, however only stages II and later are shown. Plots represent the percentage of colonies that contained each stage. *n* = 5 or more for each date at each site. Note that colonies can simultaneously contain gametocytes of different stages. The lunar cycle is shown below the x-axis, as well as a black bar that represents expected spawning dates 6–9 days after full moon in August 2011.

**Figure 4:**
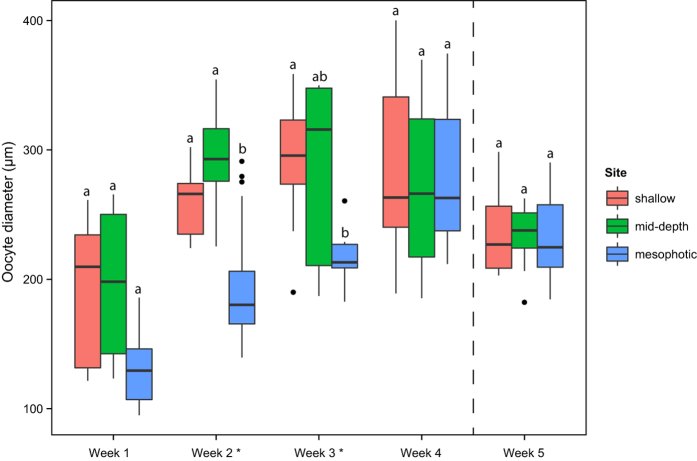
Boxplot of *O. faveolata* oocyte/ova diameters measured from histological sections from each site each sampling week. Upper and lower hinges correspond to the first and third quartiles, bars correspond to medians and whiskers extend to the highest and lowest values within 1.5 IQR (inter-quartile range). Data beyond whiskers are outliers represented as dots. Significant differences between sites were found in weeks 2 and 3 (multiple comparisons from linear mixed effect models, step-wise adjusted; see Table 1 for more information and exact adjusted p-values). The dotted line denotes that spawning was expected between weeks 4 and 5. Oocytes (likely stage III) were retained beyond expected spawning in August at all sites.

**Figure 5:**
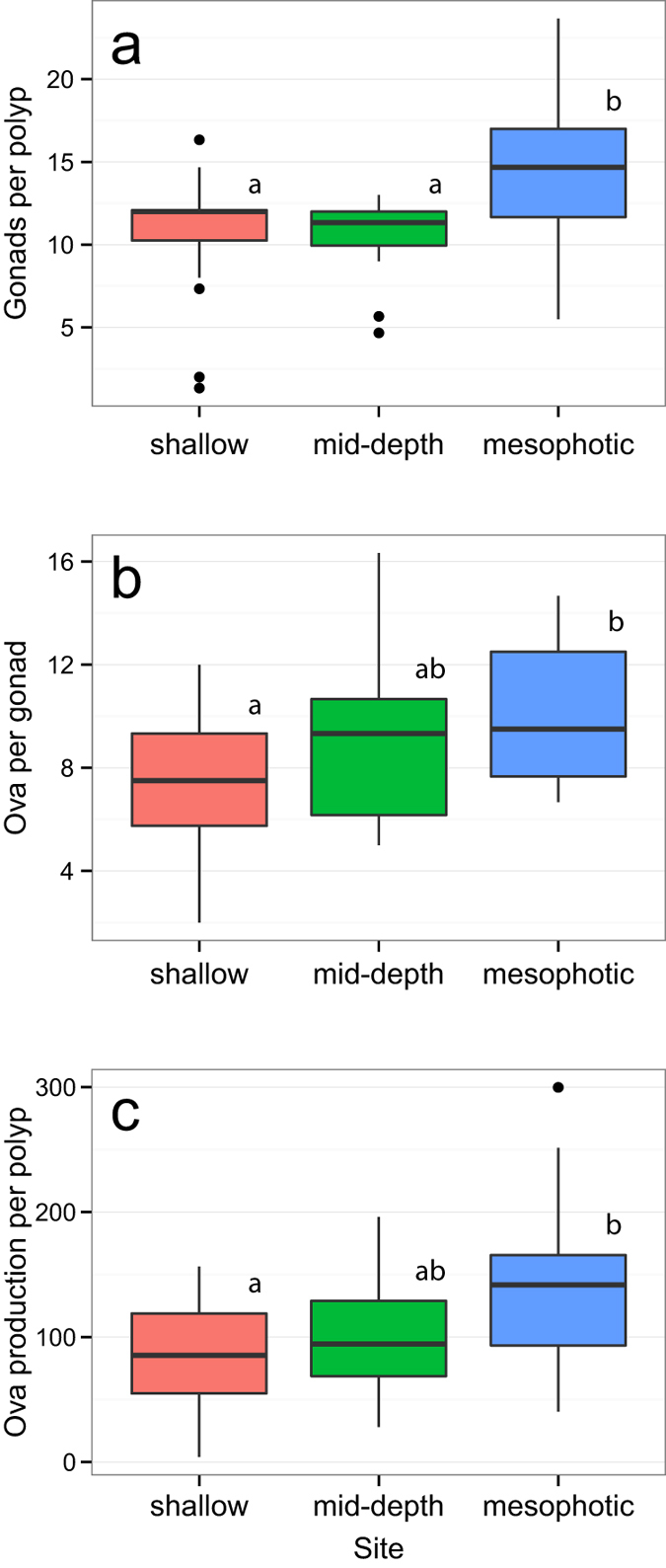
*O. faveolata* fecundity estimates and comparisons between 2011 study sites. The number of (**A**) ripe gonads and the number of (**B**) oocytes/ova per gonad were estimated for three polyps per sample. Ova production per polyp (**C**) is the product of the number of ripe gonads multiplied by the number of oocytes/ova per gonad. Data was pooled from sampling weeks 1 through 4. Comparisons were made with Kruskal-Wallis ANOVAs and the Bonferroni post-hoc method to arrive at adjusted p-values. Significant results are noted using lower-case letters in each boxplot (p < 0.05). See Fig. 4 for explanation of boxplot.

**Figure 6:**
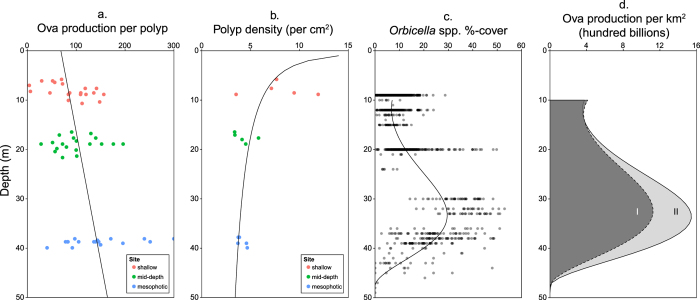
Regressions over depth of polyp fecundity, polyp-spacing, *Orbicella* spp. coral cover, and the resulting product which estimates the number of ova produced over a 1 km^2^ unit reef over depth. (**a**) A significant positive linear relationship was found between per polyp ova production and depth (p = 0.001, R^2^ = 0.18). (**b**) Mid-depth and mesophotic corals were found to have similar polyp-spacing, whereas shallow corals had significantly higher polyp densities (one-way ANOVA, p = 0.012, N = 15). A significant negative linear relationship was found after log transformation (p = 0.014, R^2^ = 0.381). (**c**) Third degree polynomial model of *Orbicella* spp. coral cover versus depth (p < 0.0001, R^2^ = 0.51). *Orbicella* spp. cover is used as a proxy for *O. faveolata* cover. (**d**) Expected *Orbicella* spp. reproductive output from hypothetical 1 km^2^ USVI reefs over depth. (I) Assumes equal polyp fecundity over depth^27^ (96 eggs per polyp^32^); (II) assumes empirically estimated depth-specific fecundity.

To test our hypotheses we selected shallow, mid-depth and MCE coral reefs off the south side of St. Thomas, United States Virgin Islands (USVI) in 2011 and 2012 (Fig. 1). This is an ideal area to study questions concerning the DRRH because the broad insular shelf and shelf edge support extensive MCEs. MCEs of the northern USVI between depths of 30–45 m are predominantly composed (>85% by cover) of members of *Orbicella* spp.^14^, the principal shallow and mid-depth structural reef-building scleractinian corals in the Caribbean^52^. We chose the species *O. faveolata* to test hypotheses concerning the reproductive potential of MCEs. This species is ideal because *O. faveolata* is a depth-generalist that can be abundant in shallow habitats and has been shown to be extremely abundant in MCEs in the USVI^14^. Its threatened status^29^ also allows for inferences regarding deep refugia.

*O. faveolata* is simultaneously hermaphroditic, with each polyp producing egg and sperm concurrently. Each colony typically spawns once a year, about one week after full moon in either August or September, and in some areas as late as October. Spawning usually lasts for under an hour, and during spawning egg and sperm bundles are extruded from the oral cavity of the polyp and float to the ocean surface where eventually they break apart and potentially cross-fertilize^32^,^33^,^53^.

### Sample collection

Preliminary sampling occurred in August 2010 to determine if mesophotic *O. faveolata* populations were gravid when expected. 70–80% of colonies sampled were reproductively active (i.e. contained gametes) at all sites from 6–39 m depth, just prior to expected spawning dates (See Supplemental Experimental Procedures and Supplementary Table S1). In July and August 2011, *O. faveolata* colonies were randomly sampled at three sites over five weeks (n ≥ 5 site^−1^ week^−1^, N = 77). The three sites included a shallow inshore site (Black Point, 5–8 m), a mid-depth off-shore island site (Flat Cay, 16–21 m), and a mesophotic site (Grammanik Bank, 37–40 m) (Fig. 1). All tissues were fixed in zinc-buffered formalin (Z-Fix) for 24 hours, rinsed in fresh water for 24 hours, and stored in 70% EtOH until processing. The surface area of each sampled coral colony was estimated to ensure that depth-fecundity relationships were not confounded by fecundity-size relationships (see Supplemental Experimental Procedures and Supplementary Fig. S4).

In 2012 partial colonies of *O. faveolata* were collected several days before predicted spawning in both August and September from a mesophotic site (35–40 m, n = 10) and a shallow site (5–10 m, n = 10) for laboratory observation. These samples were kept in temperature-controlled (27 °C+/−1 °C) flow-through seawater tables and were exposed to near-natural light cycles. In the evenings throughout the potential spawning window, these partial colonies were observed for spawning behavior in isolated glass jars. Additionally, video systems were deployed in August and September 6–9 days after full moon to capture video of *in situ* spawning behavior in an MCE. Video systems were built using two GoPro HD (http://www.gopro.com) cameras and external lights mounted on a weighted plastic frame. The system was deployed from a boat using a Sea Viewer drop camera to guide the placement on top of an *O. faveolata* colony. The drop camera was subsequently pulled free and the system was allowed to record through the potential spawning window on a given night.

### Histological preparations

Samples were decalcified using 10% HCl solution and 0.5 g/L EDTA. This solution was replaced every 24 hours until all calcium carbonate had been dissolved, after which tissue was stored in 70% EtOH. A tissue processor (Sakura Tissue-Tek II) was used to dehydrate and parafinize tissues. Parafinized tissue was arranged for cross and longitudinal sectioning and embedded in paraffin blocks using a tissue embedding station (Sakura Tissue-Tek TEC). Blocks were then sectioned on a Leica RM2235 microtome with 4 micron thickness at five depths, beginning just below the oral opening, with subsequent sections occurring every 100 microns. Sections were arranged on microscope slides stained using a modified Heidenhain’s aniline blue stain.

### Reproductive characteristics

*O. faveolata* histological sections were analyzed for (1) presence/absence of male and female gonads and gametes of each reproductive stage^30^ (complementary staging techniques are also becoming more commonly used^54^); (2) the fecundity, or number of oocytes or ova per gonad, and the number of gonads per polyp^27^,^33^,^55^; as well as (3) the diameter and condition of oocytes. Observations were made using both standard light microscopy and an Olympus VS120-S5 digital slide scanner. Measurements were made using Fiji software (ImageJ).

The number of polyps per surface area was estimated for a subsample of colonies in order to account for increased polyp spacing with depth^56^,^57^. A white light 3D scanner (3D3 Solutions HDI Advance) was used to digitize samples in high resolution. Polyps were enumerated on the scans in Leios 3D data processing software. Those digital surfaces were smoothed to find a basal surface area (Supplementary Fig. S5).

*O. faveolata* fecundity per cm^2^ (*F*) was estimated as the product of:

*F* = (eggs * gonad^−1^) * (gonads * polyp^−1^) * (polyps * cm^−2^)

In order to derive depth-specific unit reef egg production, estimates of percent coral cover were calculated from diver video transects performed at each site from 2001–2012^58^. *Orbicella* spp. coral cover was used as a proxy for *O. faveolata* coral cover since it is often difficult to distinguish *Orbicella* species from video at mesophotic depths. It is expected that *O. faveolata* cover trends with total *Orbicella* species cover in this area. This data was plotted against depth (square root-transformed) and a third-order polynomial model was fitted to the result. The products of equation 1 were then multiplied by estimated coral cover at each depth to approximate the number of eggs per 1 km^2^ unit reef as a function of depth.

## Additional Information

**How to cite this article**: Holstein, D. M. *et al.* Fertile fathoms: Deep reproductive refugia for threatened shallow corals. *Sci. Rep.* **5**, 12407; doi: 10.1038/srep12407 (2015).

## Supplementary Material

Supplementary Information

Supplementary Video S1

## Figures and Tables

**Table 1:** Results of multiple comparisons among sites (depths) from linear mixed effects models of *O. faveolata*oocyte/ova diameters within weeks.

	**Post-hoc comparison**	**Observations**	Adjusted *P*
Week 1	shallow—mid-depth	108	0.977
	mid-depth—mesophotic		0.209
	shallow—mesophotic		0.363
Week 2	shallow—mid-depth	120	0.1166
	mid-depth > mesophotic		<0.0001*
	shallow > mesophotic		0.0047*
Week 3	shallow—mid-depth	105	0.9804
	mid-depth—mesophotic		0.0621
	shallow > mesophotic		0.0208*
Week 4	shallow—mid-depth	147	0.739
	mid-depth—mesophotic		0.904
	shallow—mesophotic		0.946
Week 5	shallow—mid-depth	102	0.999
	mid-depth—mesophotic		0.947
	shallow—mesophotic		0.957

Note that spawning was evident between weeks 4 and 5. See also Fig. 4. Oocyte observations were nested within colonies to avoid pseudoreplication. Asterisks denote significant comparisons and a rejection of the null hypothesis of equal oocyte diameter between depths with an alpha of 0.05. p-values are step-wise adjusted.

**Table 2:** Fecundity estimates for each site in 2011.

**2011**	**Site**		**Mean (±SE)**	
	**Black Point (shallow)**	**Flat Cay (mid-depth)**	**Grammanik Bank (mesophotic)**	**Overall** ***P***
Depth (m)	5–10	15–22	35–40	
Colonies	20	20	19	
Gonads*polyp^−1^	10.78 (±0.83)^a^	10.58 (±0.48)^a^	14.39 (±0.97)^b^	<0.01*
Ova*gonad^−1^	7.48 (±0.59)^a^	9.19 (±0.72)^ab^	9.91 (±0.60)^b^	0.046*
Ova*polyp^−1^	87.05 (±10.10)^a^	99.72 (±10.15)^a^	144.43 (±15.91)^b^	0.021*

Data was pooled from weeks 1 through 4 of sampling (prior to expected spawning). Means and standard deviations are shown, however non-parametric significant differences between sites are noted by lowercase letters (Kruskal-Wallis ANOVA/Bonferroni method, alpha = 0.05).
