# Supplementary material for: Fertile fathoms: Deep reproductive refugia for threatened shallow corals
Source: Sci Rep. 2015 Jul 21;5:12407. doi: 10.1038/srep12407 (PMC4508916; doi:10.1038/srep12407)
Supplement: Supplementary Information [file srep12407-s1.pdf]

Fertile fathoms: Deep reproductive refugia for threatened shallow corals

Daniel M. Holstein*, Tyler B. Smith, Joanna Gyory and Claire B. Paris

Supplemental Information

Supplemental Experimental Procedures

Further information on 2010 tissue sampling and analysis

In 2010 samples were taken from five reef sites on August 26-28, several days prior to the date of expected spawning in August. Sites ranged in depth from 6-43 m (n >= 10*site^{-1}, N = 79, Table S1). In 2010 the percentage of *M. faveolata* colonies that were reproductively active was between 70% and 80% for all sites visited but one (Table S1). For the most part, mesophotic sites appeared just as, if not more reproductively active than shallow and mid-depth sites. Over half of colonies at the deepest site visited (43 m) were reproductive, but the site was nearly 20% less reproductive than any other. This could have been due to colonies at this depth experiencing a deep low-light light threshold; however more than half of the population was found to be putting some proportion of metabolic energy into reproduction. It is also possible that the study was limited by sample size in this instance (n=10).

Colony surface area estimates

Colony surface area was estimated as half the approximated surface area of a three-dimensional scalene ellipsoid using the longest diameter, perpendicular diameter and the height of the colony (from the Thomsen approximation[1]):

$$Surface\ Area \approx 4\pi\left(\frac{1}{3}*\left(\left(\frac{a}{2}\right)^{p}\left(\frac{b}{2}\right)^{p}+\left(\frac{a}{2}\right)^{p}(c)^{p}+\left(\frac{b}{2}\right)^{p}(c)^{p}\right)\right)^{\frac{1}{p}}\Big/2$$

Where a is the longest diameter of the colony, b is the diameter of the colony perpendicular to a, and c is the height of the colony. p is a constant equal to 1.6075. This method should theoretically be a better estimator of coral surface area than a two-dimensional ellipse or an average hemisphere, particularly when making comparisons between shallow and mesophotic coral colonies that have different morphologies (Fig. S5).

Supplemental Figures

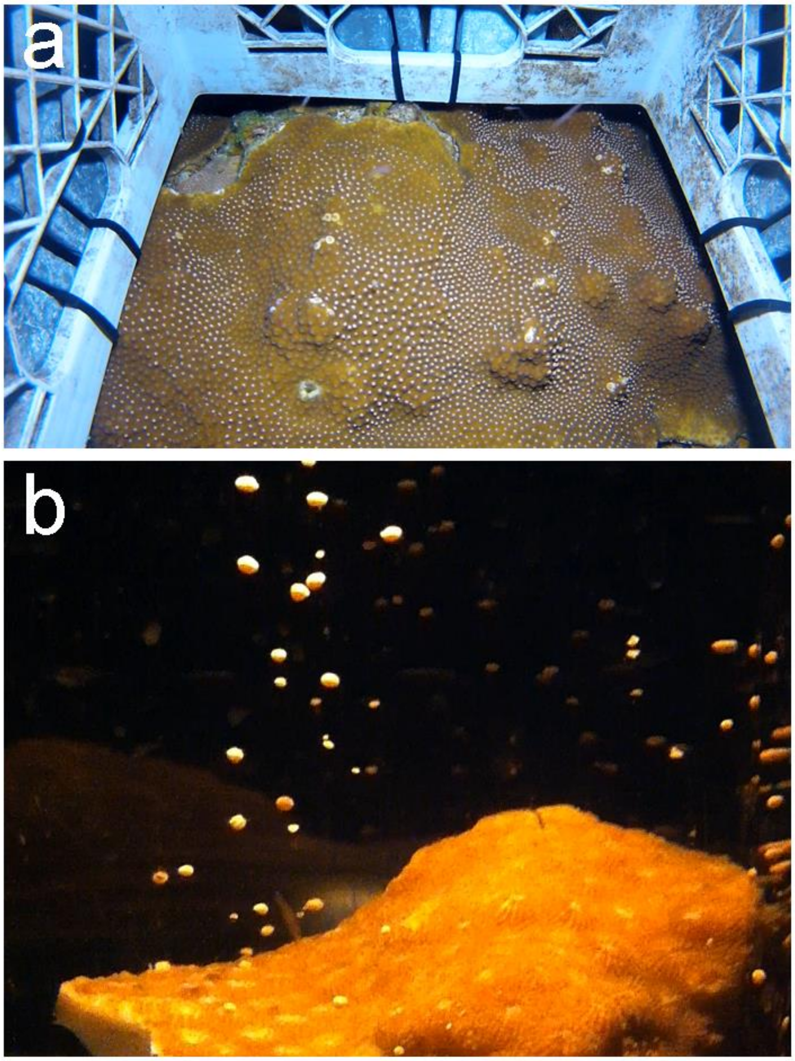

Fig. S1. Coral spawning observations. (a) A large *O. franksi* colony setting at ~38m at 20:50 one week after full moon in September 2012. The colony underwent a whole-colony spawn. *Video is available.* (b) Spawning of an *O. faveolata* coral fragment observed in the laboratory on the same evening, at 20:45. *Video is available.*

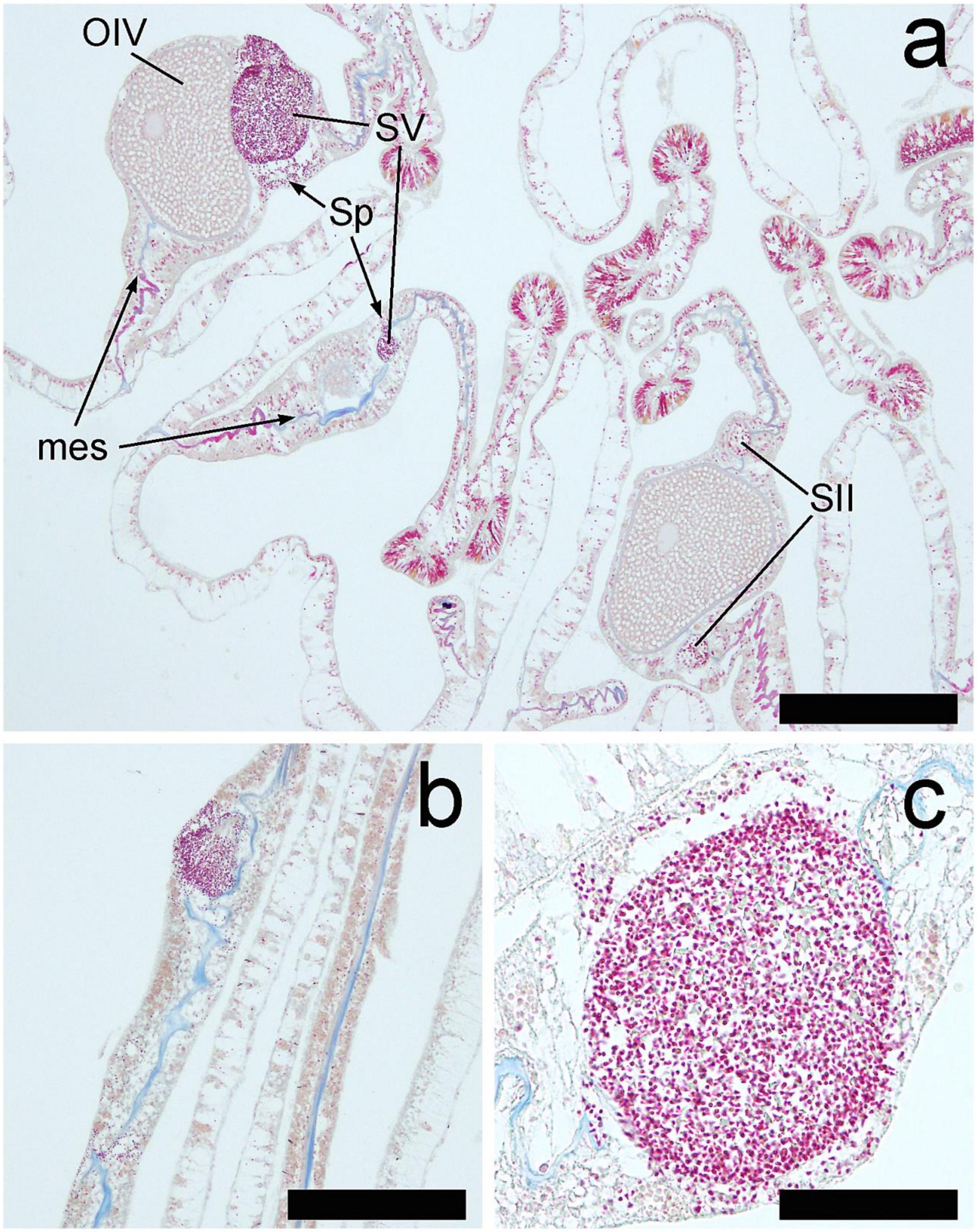

Fig. S2. Histological evidence of spawning in *O. faveolata*. (a) 'Wasted' or 'loose' mesenteries (mes), remnant stage V spermaries (SV) and free spermatozoa in the mesentery (sp) are indicative that the polyp has undergone spawning. In this case, there are also remnant stage IV

ova, and evidence of developing stage II spermaries. (b) Another example of ‘wasted’ mesenteries, a remnant stage V spermary and free spermatozoa. (c) A close-up of a remnant stage V spermary and free spermatozoa not confined to the spermary (bar, a = 200 µm; b = 200 µm; c = 50 µm).

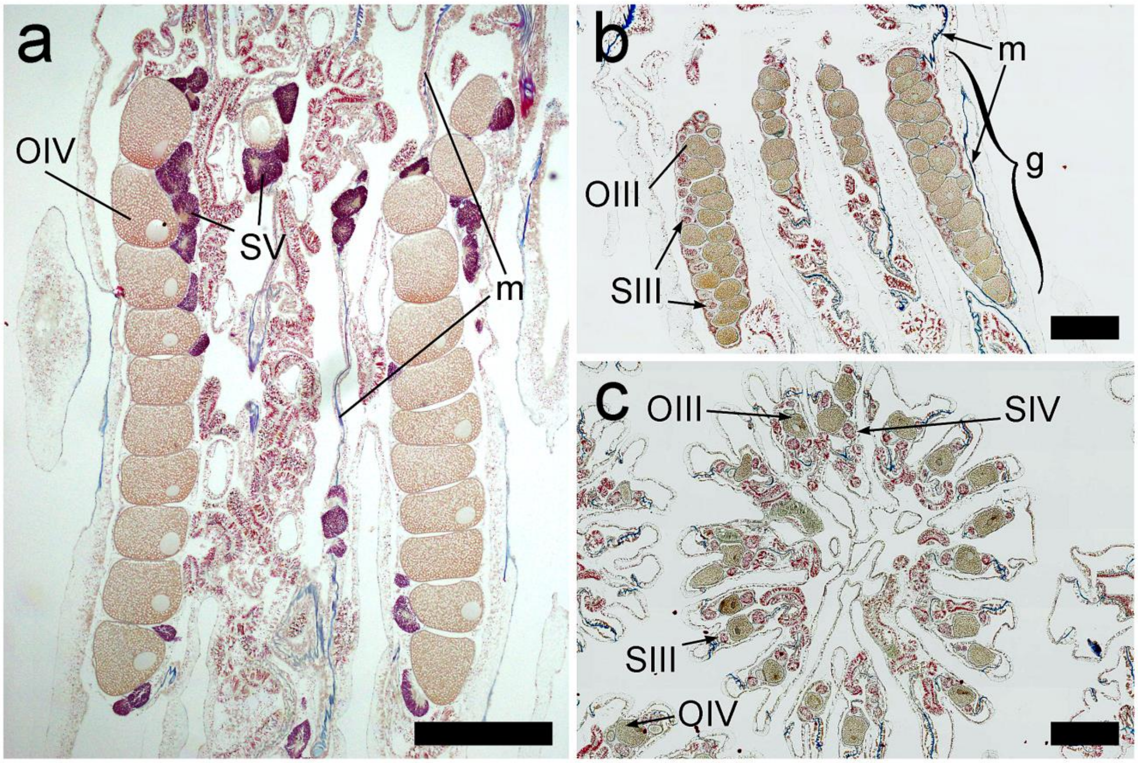

Fig. S3. (a) A typical longitudinal section showing stage IV ova (OIV) and stage V spermaries (SV) arranged in gonads. The ova and spermaries are within the mesoglea, which is stained blue (m). (b) An example of highly fecund gonads (g) containing many more than 8-12 stage III oocytes (OIII) surrounded by stage III spermaries (SIII). In this example the gonad can be seen inside the mesoglea (m), attached to the mesenterial wall. (c) An example of a polyp containing many more than 12 gonads in cross-section, in this case at least 20. Gametocytes of different stages can be found within the same colony, the same polyp, and even the same gonad simultaneously.

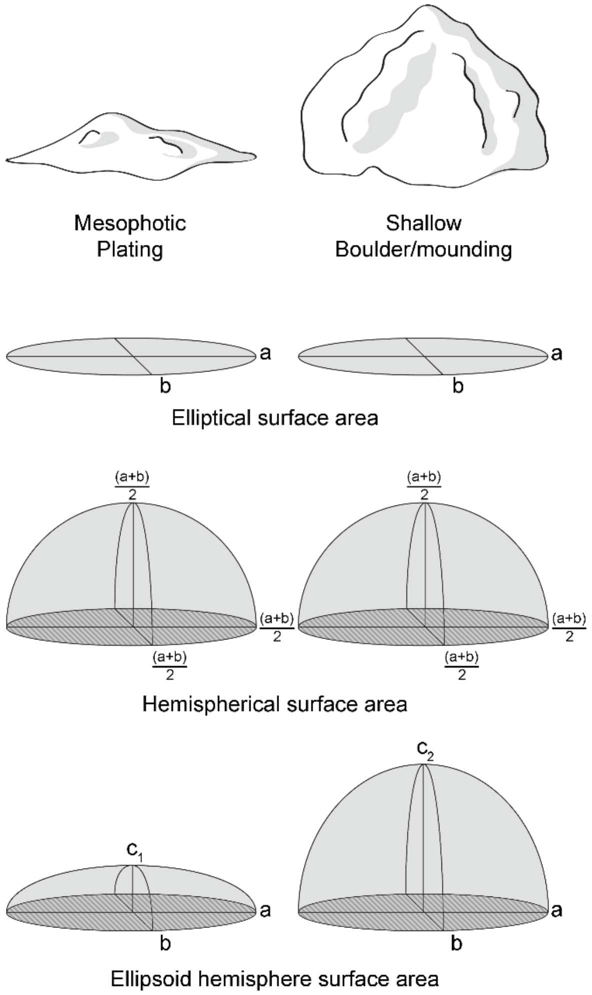

Fig. S4. An illustration of the benefit of utilizing the surface area of a scalene ellipsoid when estimating coral colony surface area. This is preferable to the use of the hemispherical surface area, particularly when $a \neq b \neq 2c$ and in the case of *O. faveolata*, which has different morphologies in different habitats, and thus a ratio of width and height is not constant.

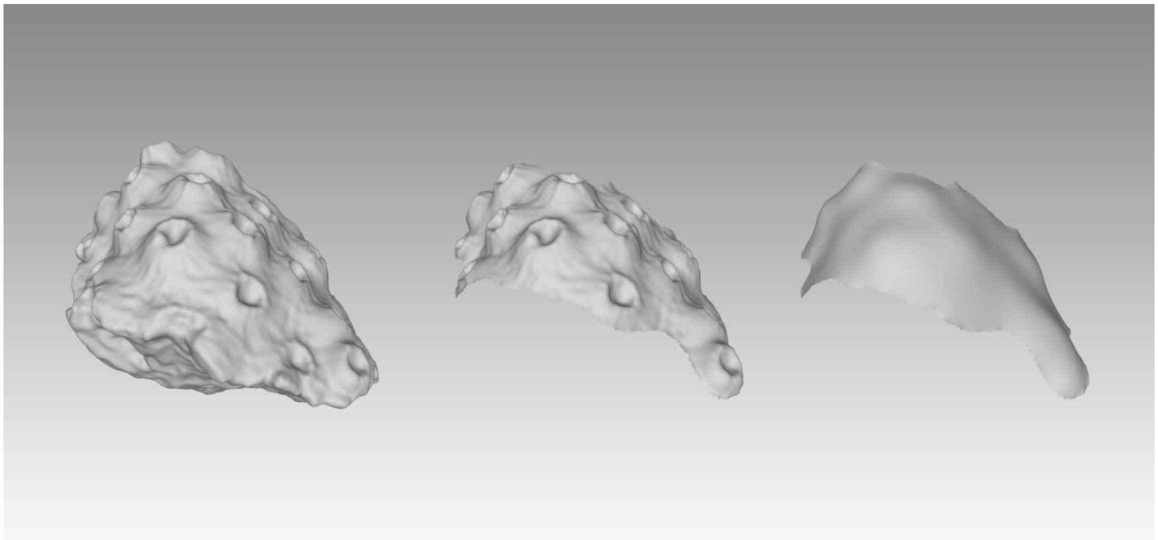

Fig. S5. Three-dimensional surface reconstruction of an *O. faveolata* coral sample taken with a hammer and cold chisel. Five samples per site (N=15) were scanned and reconstructed, and the coral surface of the reconstruction was isolated and smoothed in order to estimate basal coral surface area. The polyps of each sample were enumerated and divided by this basal surface area, and the resulting polyp densities were compared between sites.

Supplemental Tables

Table S1. Percent colonies found to be reproductive during preliminary sampling in 2010. Sampling occurred over three days, several days prior to expected spawning in August.

2010	Site				
	Flat Cay	S. Capella	College Scholl	Hammerhead Scholl	Grammanik N. Bank
Depth (m)	6	22	34	39	43
n	18	21	10	19	11
% colonies reproductive	72.22%	76.19%	80%	73.68%	54.55%

Supplemental References

1. Xu D, et al. (2009) The ellipsoidal area ratio: an alternative anisotropy index for diffusion tensor imaging. *Magn Reson Imaging* 27(3):311–23. Available at: http://www.pubmedcentral.nih.gov/articlerender.fcgi?artid=3575168&tool=pmcentrez&rendertype=abstract [Accessed April 25, 2013].
